# Primate carcass ecology in the Urema Rift, Mozambique

**DOI:** 10.1007/s10329-026-01275-y

**Published:** 2026-07-20

**Authors:** Jacinto Mathe, René Bobe, Mércia Ângela, António Tonecas, Paola Bouley, Susana Carvalho

**Affiliations:** 1 Primate Models for Behavioural Evolution Lab, Institute of Human Sciences, University of Oxford, Oxford, UK; 2 Department of Science, Gorongosa National Park, Goinha, Mozambique; 3 ICArEHB-Interdisciplinary Center for Archaeology and Evolution of Human Behaviour, Universidade do Algarve, Campus Gambelas, 8005-139, 13 Faro, Portugal; 4 CIBIO/InBIO, Centro de Investigação em Biodiversidade e Recursos Genéticos, Universidade do Porto, Campus de Vairão, Vairão, Portugal

**Keywords:** Environmental variables, Gorongosa landscapes, Human evolution, Bone assemblages, Faunal community

## Abstract

Primates constitute an integral part of many African ecosystems. In some areas, primate species can be locally very abundant, while in others they are rare or absent. Our central question is: does the abundance of primate bones (or carcasses) reflect the abundance of local living populations? Past bone surveys in Amboseli National Park, Kenya, and Virunga National Park, Democratic Republic of Congo, revealed a positive correlation between carcass abundance and living mammal communities, with a notable exception: a low abundance or absence of primate carcasses when compared to other medium-sized and large mammals. Recent research conducted at Gorongosa National Park, Mozambique, revealed a similar discrepancy between the abundance of primate carcasses and mammalian live census data. This study aims to understand this mismatch between live and dead by examining the influence of ecological factors on primate carcass occurrence and bone distribution in the modern rift ecosystem of Gorongosa National Park. To address these issues, we investigated whether (1) landscape type, proximity to roads, and water sources (pans, rivers, and lakes) predict primate carcass occurrence relative to other mammal taxa, and (2) the accumulation of primate bones significantly differs from that of other mammals in annually flooding and non-annually flooding landscapes (broadly speaking, in the floodplain vs. higher areas). We analysed 26 bones from 18 chacma baboon individuals (*Papio ursinus*) and 255 bones from 20 other mammal species. Generalised Linear Model (GLM) outputs suggest that permanent water sources such as rivers and lakes are key drivers of primate bone accumulation (p < 0.05). A Fisher’s Exact Test indicates that primate bones are deposited more frequently in Rift Valley Riverine & Floodplain landscapes (p < 0.05). Given that baboons regularly cross rivers in Gorongosa, these findings also suggest that crocodile predation may play an important role in determining where baboon carcasses ultimately accumulate, potentially contributing to the observed mismatch between living and dead communities. These findings have important implications for our understanding of primate bone accumulations in the African fossil record, where some palaeontological sites contain abundant primate fossils whereas others preserve few or none.

**Supplementary Information:**

The online version contains supplementary material available at https://doi.org/10.1007/s10329-026-01275-y.

## Introduction

Primates constitute an integral part of many African ecosystems, both modern and ancient. In some areas, primate species can be locally very abundant, while in others they are rare or absent. Our central question is: does the abundance of primate bones (or carcasses) in the landscape reflect the abundance of local living populations? For example, live census data from places such as Gorongosa National Park (GNP), Mozambique, indicate that cercopithecoid primates such as *Papio ursinus* are among the most numerous mammals in the region, surpassed only by a couple of species of highly abundant antelopes (Stalmans and Peel 2024). Nevertheless, recent osteological surveys at Gorongosa suggest that the abundance of primates is lower than expected based on the living population numbers. Bone surveys from other parts of Africa have similarly yielded relatively low numbers of primates compared to other mammalian taxa. For instance, Behrensmeyer’s long-term taphonomic study at Amboseli National Park (ANP), Kenya, recovered only a few baboon bones over a 50-year period (Behrensmeyer 1993, 2007; Behrensmeyer et al. 1979; Western and Behrensmeyer 2009), even though baboons were initially abundant in the park. Baboon abundance at Amboseli declined markedly from around 2,500 individuals in 1963–1964 to just 125 individuals by 1979 (Altmann et al. 1985). In more recent years, the main baboon study groups at Amboseli have consisted of approximately 350 individuals, representing a minimum estimate for the park’s population (Alberts et al. 2012). Thus, the scarcity of baboon bones in Behrensmeyer’s study is puzzling. A similar pattern was observed in a survey conducted at Virunga National Park (VNP) in the Democratic Republic of Congo, which did not yield a single primate bone among roughly 4,500 osteological specimens, even though baboons were common in the study area (Tappen 1995). What factors account for the abundance or scarcity of primate bones in regions where the animals are clearly present? Answering this question may help clarify patterns of preservation in the primate fossil record.

Beyond the many variables that are likely to affect differential distribution in bone assemblages (e.g., life history, demographics, body mass, bone abundance and surrounding tissue) ecological factors are thought to influence and partially explain aspects of bone preservation and deposition (Lam and Pearson 2005; Pickering and Carlson 2002; Tappen 1995). Previous studies focusing on neotaphonomy and modern bone assemblages in the East African Rift System (hereafter EARS) are limited and focused on a few ecological variables, i.e., presence of water and trees (Tappen 1992, 1995). For example, Tappen’s research in VNP found that more bones accumulated in the plateau grasslands and riverine habitats than in other habitat types such as hills and valleys. In Tappen’s habitat categories, plateau is an open grassland with abundant ungulates, particularly dense herds of kob (*Kobus kob*) and reedbuck (R*edunca redunca*); the riverine zone has higher tree and bush density than the plateau but does not form a dense gallery forest; hills are mainly covered in grassland, with ridges of trees and bushes; between the hills there are valleys, which are also characterized by trees and bushes. Tappen (1995) found no evidence of increased bone deposition near permanent water bodies or ephemeral water holes (Tappen 1995). In Gorongosa, the abundance of primate carcasses did not match the abundance of living primates estimated from the recorded number of troops (Stalmans and Peel 2024). Data captured by camera traps and biennial aerial surveys places baboons as the second most abundant medium-sized mammal species in the park. However, in osteological surveys baboons (*Papio ursinus griseipes*) were the fourth most abundant species, suggesting lower bone recovery or preservation for this primate species in relation to the living population. Despite their abundance and wide distribution in the Gorongosa ecosystem, vervet monkeys (*Chlorocebus pygerythrus*) were not counted in aerial surveys (Beardmore‐Herd et al. 2025; Stalmans and Peel 2016, 2020, 2022; Stalmans et al. 2018), and they are captured at low frequencies by a camera trap grid that has been ongoing since 2016 (Gaynor et al. 2019). No skeletal remains were found in osteological surveys in GNP (Mathe et al., in review). Moreover, the biennial aerial survey data show that baboon troops recorded in aerial surveys between 2016 and 2022 are widely distributed across the surveyed block area. A smaller number of troops was found in areas that flood annually, while larger numbers were found in areas that do not flood annually (Stalmans and Peel 2016, 2020, 2022; Stalmans et al. 2018). Baboon data on the living population are from four aerial counts sampled years 2016, 2018, 2020, and 2022, which overlap the period of osteological surveys (2016–2022) (Stalmans and Peel 2016, 2020, 2022, 2024; Stalmans et al. 2018).

### Behaviour and ecology of chacma baboons (*Papio ursinus*)

Baboons prioritize safe habitats over food-rich areas, foraging near refuges like trees and cliffs to minimize predation risk, which may also affect where skeletal remains accumulate (Hill and Cowlishaw 2002). As omnivores, their diet includes animals, fruits and pods (including seeds), flowers, leaves and subterranean items (e.g. roots and corms) (Cowlishaw 2013; Hill and Cowlishaw 2002). While they prefer daily drinking, they can survive extended periods without water, increasing mortality risks during droughts (Hamilton 1985). Seasonal dietary shifts, particularly during food shortages, influence survival, especially for juveniles learning from adults (Kingdon 2013a). The home range expansion due to baboon movement across the year varies according to seasonality, weather, and food resource availability (Johnson et al. 2015). These factors influence where the baboons live and die, and thus where their bones may occur.

Chacma baboons (*Papio ursinus*) live in stable troops of 22–79 individuals with female-biased sex ratios (Cowlishaw 2013; Henzi 1999; Kingdon 2013a). In Gorongosa NP there are more than 200 baboon troops, two of which are being intensively studied. The ‘woodland troop’ has 88 individuals while the ‘floodplain troop’ has 37 individuals (Hammond et al. 2022; Lewis-Bevan et al. 2025). At ANP, the troop size ranges from 35 to 65 individuals (Bulger and Hamilton 1987). Males disperse at adulthood, while females are philopatric. Males may commit infanticide upon joining a group to induce oestrus in females, who counter this by mating with multiple males and forming protective friendships (Palombit et al. 2001).

### Mortality in baboons

Understanding the processes that generate modern primate death assemblages is essential for interpreting fossil occurrences. For example, a long-term study of captive baboons showed that mortality resulted from a wide range of causes, including stillbirth, gastrointestinal disease, haemorrhage, trauma, seizures, and respiratory infections, highlighting the complexity of factors that can shape primate skeletal representation before fossilization (Dick et al. 2014a).

In nature, the main cause of mortality in baboons is predation, mostly by leopards, occasionally by lions, hyenas, crocodiles, jackals, eagles, cheetah, domestic dogs, pythons, and wild dogs (Cheney et al. 2004). Adult males often defend groups, taking on high-risk roles during travel (Cheney et al. 2004). Baboons also suffer from parasites, including ticks, and diseases such as bacterial yersiniosis. The most common diseases reported in captivity are haemorrhage and colitis, and these vary across sex and age (Dick et al. 2014b). In the wild, mortality is highest among infants, mainly due to infanticide and predation, and can vary with group size and rank (Dick et al. 2014b). Starvation has been documented in extreme cases, disproportionately affecting females, juveniles, and infants (Hamilton 1985). Seasonal dispersion and predictable travel routes increase predation and mortality risks (Cheney et al. 2004).

Other predators present at GNP may also contribute to primate mortality, although usually at lower frequencies. Lions occasionally prey on baboons, particularly when troops are foraging terrestrially in open habitats, whereas spotted hyenas may kill vulnerable individuals or scavenge carcasses (Busse 1980; Isbell 1994). Crocodiles represent an important source of mortality at river crossings and water points, especially for juveniles and dispersing individuals (*Erin Phillips, person communication*). Large raptors, particularly crowned eagles, are significant predators of smaller primates in African forests and can account for substantial mortality among monkey populations (Cheney et al. 2004; Palombit et al. 2001; Seike 2022).

### Baboons as models for early hominin ecology and behaviour

Palaeoanthropological research helps us to understand the ecology and behaviour of early hominins (Clarke et al. 2021). However, there are limitations regarding the preserved physical remains used for palaeoanthropological interpretations (King 2024). The use of baboons as models for studying early hominin ecology and behaviour is rooted in three main functional similarities: cosmopolitan distribution, flexibility in adaptation to different environments, and body mass. Both hominins and baboons inhabit wide and diverse habitats, from woodlands to grasslands and cold highlands, and are adapted to drastic cycles of climate change with great flexibility in their physiology and behaviour (Arráiz et al. 2017; Bobe et al. 2020; Magill et al. 2016). The flexibility and adaptability to diverse environmental conditions is a shared characteristic between hominins and baboons (King 2024). This adaptation allows them to navigate a variety of habitats in search of food, climb trees, and tolerate different levels of stress. Body mass is slightly different between the two taxa, as early hominins weigh between 25 and 75 kg, and baboons range from 15 to 35 kg, with significant overlap, making this factor one of their shared traits (Fischer et al. 2019; Simpson et al. 2019). Both are considered medium-sized when compared to large mammals like elephants or hippopotamus and considered large-sized in comparison to most primates, except for chimpanzees, bonobos, and gorillas (King 2024). Despite aspects of body mass and canine teeth regarding sexual dimorphism, which varies across baboon species but is relatively large in all of them and not as evident in early hominins, baboons continue to be considered as models for understanding the ecology and behaviour of early hominins, helping to shed light on hominin evolution and fossil deposition in different ecosystems (King 2024).

In this study, we examine whether (1) the landscape type, proximity to water bodies (pans, lakes and rivers) and roads predict baboon (hereafter primate) carcass occurrence compared to other mammal taxa, and (2) the abundance of primate bones is significantly different from other mammal taxa in annually flooding and non-annually flooding landscapes. We predict that (1) the likelihood of primate carcass occurrence will be lower than that of non-primate carcasses in both, annually flooding and non-annually flooding landscapes, (2) primate carcass occurrence will be lower close to water sources and roads than further away and (3) primate bone abundance differs significantly from that of non-primate carcasses across the two GNP landscapes, with the annually flooding landscape harbouring a higher bone abundance than non-annually flooding landscapes. This pattern is driven by behavioural and ecological factors unique to primates, such as reduced water dependency (Pontzer et al. 2021) and higher risk perception (Hammond et al. 2022, 2025), which lower the bone abundance of primates relative to other mammal taxa in these environments. These factors may affect bone preservation and deposition rates and carcass accumulation in inaccessible and low-elevation locations. These predictions aim to provide a comprehensive understanding of the factors influencing primate carcass occurrence and bone distribution in GNP. An understanding of the variables that influence the occurrence and distribution of primate bones in modern landscapes can help us understand the variability of primate fossils in palaeontological contexts (Bobe 2011; Nel et al. 2021).

## Methodology

### Study site

**Fig. 1:**
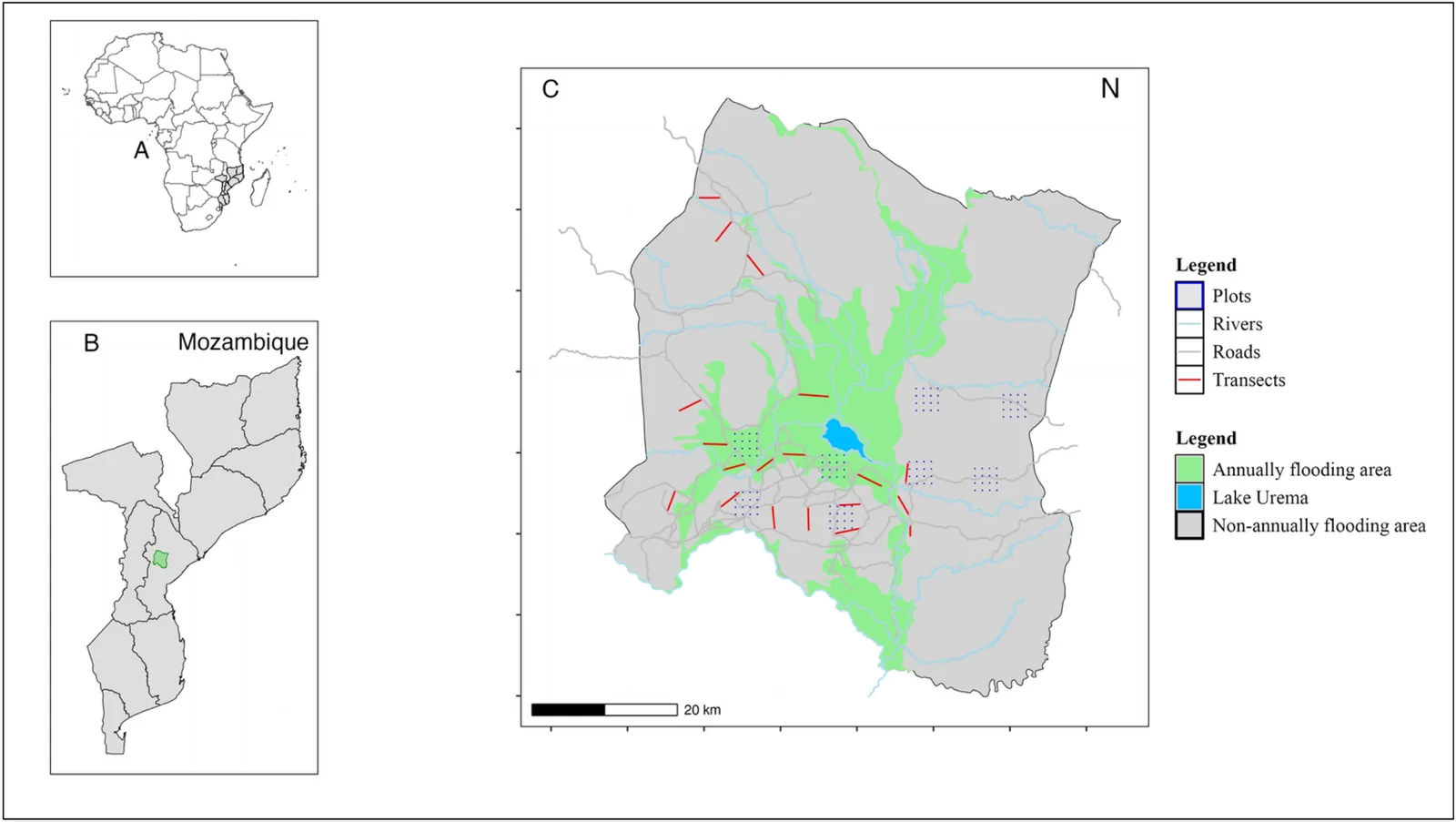
Examples of annually and non-annually flooding landscapes of Gorongosa National Park, Mozambique

This research was conducted in Gorongosa National Park (GNP) in central Mozambique. GNP is a protected area of approximately 4000 km^2^ bounded by the Pùngué River in the south and Zambezi River in the north (Fig. 1), at the southern end of the East African Rift System (EARS) (Stalmans et al. 2019; Tinley 1977). GNP has diverse landscapes (Stalmans and Beilfuss 2008) that sustain almost 8000 identified species of flora and fauna (Gorongosa Biodiversity Database) (Gorongosa National Park 2024). Containing the last portion of the EARS dated to 3MA (+/−1MA) (Habermann et al. 2019; Macgregor 2015) and an extensive cave system, Gorongosa is a valuable analogue for studying environmental and ecological variables that have determined fossil site formation in eastern Africa (Bobe et al. 2023, 2020; Bobe and Carvalho 2019).

Historically, GNP is known as an example of wildlife rebounding after losing almost 90% of its wild animal population due to the Mozambican Civil War (1977-1992), during which fauna was hunted for sustenance and military equipment/ivory exchange (Daskin et al. 2016). This war devastated a place that was known as a sanctuary for a variety of large mammals, including buffalo, blue wildebeest, zebra, waterbuck, impala, and hippopotamus (Stalmans et al. 2019). The park’s revival commenced in 2008 with the establishment of the Gorongosa Restoration Project by a non-profit organisation. This initiative, which signed a 20-year co-management agreement with the Mozambican Government, concentrated on conservation, science, and human development. The project was extended for an additional 25 years in 2018 (Stalmans et al. 2019).

Geographically, GNP is between −19.44799 and −18.01795 of latitude and within 33.91316 and 34.89479 of longitude. On the west GNP is bounded by Gorongosa Mountain and on the east by the Cheringoma Plateau. Gorongosa Mountain was proclaimed part of GNP in 2010 and is home to many endemic species. It is also where many rivers start and flow to Lake Urema, the largest body of permanent water within the park, as well as the many water courses within GNP landscapes. The climate is predominantly tropical, with a dry season spanning from May to November and a rainy season from December to April (Tinley 1977). Annual rainfall ranges from 700 to 900 mm in the Rift Valley floor, reaching higher levels of over 2000 mm on Gorongosa Mountain (McWethy et al. 2016; Müller et al. 2012). Every year, GNP floods in the rainy season and about 40% of the park is underwater (Tinley 1977).

Geologically, GNP hosts the southern portion of the East African Rift System which is generally characterised by alluvial deposits with colluvial material deposited at the base of the Cheringoma Plateau. The Cheringoma Plateau consists of grauwackes and limestone with impermeable horizons and soils with low pH and high phosphorus content, which contribute to the fast bone weathering. Between the Rift Valley and Gorongosa Mountain, there are the Midlands, mostly underlain by gneisses and pegmatites with many dolerite dykes and sandy and fersiallitic soils, which also contribute to quick bone weathering due to dissolving hydroxyapatite, the main bone component (Stalmans and Beilfuss 2008).

The hydrology of the park consists of a multitude of rivers and streams. These water sources start from Gorongosa Mountain, Midlands and Cheringoma Plateau and end in the Rift Valley with Lake Urema as the centre of water accumulation and draining its water to the Indian Ocean through the Pùngué River (Stalmans and Beilfuss 2008). During the dry season, some pans (wetlands with waterlogged soils and/or aquatic vegetation) maintain water until the next rainy season while others are seasonal. Permanent pans are well-mapped and described in the park.

**Fig. 2:**
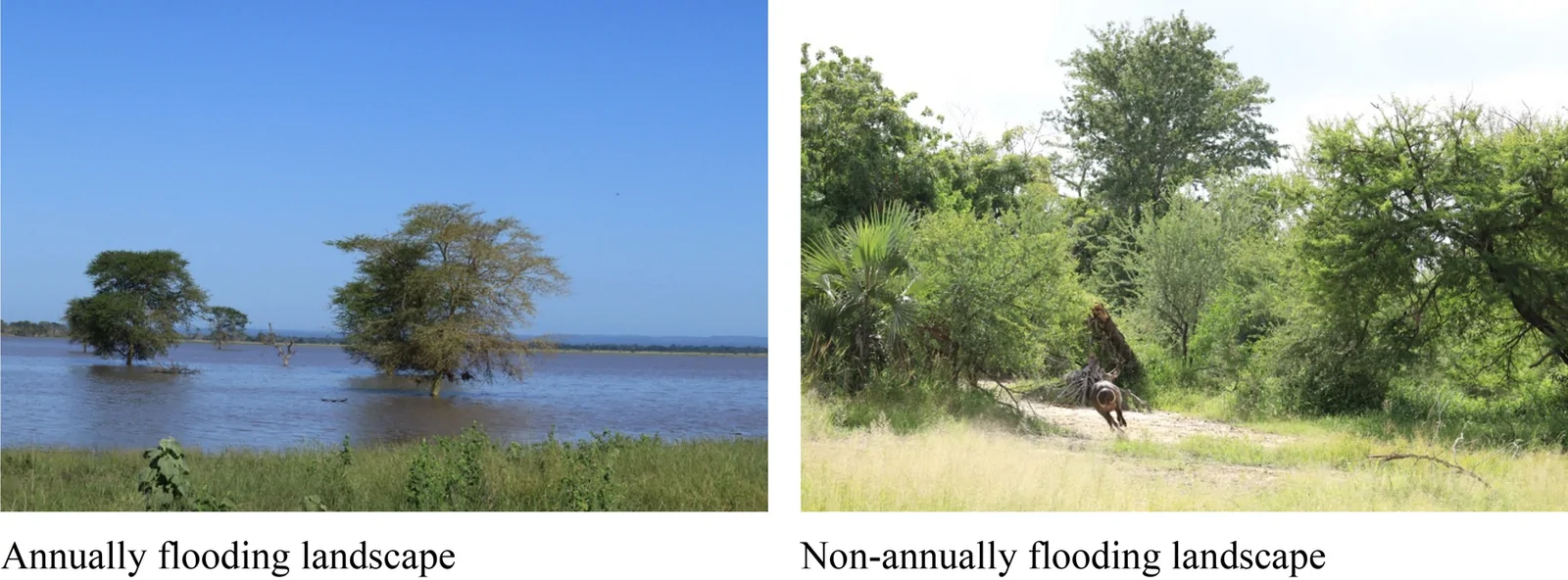
Map of Gorongosa National Park, Mozambique, showing the locations of plots and transects used for bone surveys (**C**). Insets show the location of Gorongosa National Park within Mozambique (**B**) and Mozambique within Africa (**A**)

Unpaved roads, locally called ‘picadas’, are used in the park for patrolling and tourism purposes. Only 20% of GNP is covered by the road network, most of which is in the south of the park close to the main camp, Chitengo, and the Community Centre of Education (Fig. 2, where roads are depicted).

Currently, GNP has over 8000 species of fauna and flora identified, among which 825 are vertebrates (Gorongosa Biodiversity Database). Among these are five primate species: grey-footed chama baboons (*Papio ursinus griseipes*), vervet monkeys (*Chlorocebus pygerythrus*), Samango monkeys (*Cercopithecus mitis erythrarchus*) and two species of galago (*Otolemur crassicaudatus* and *Paragalago granti*). Of the approximately 230 troops of baboons recorded in the park, six individuals of the two troops (three individuals per troop) were collared and are being monitored since 2018.

The reintroduction of fauna in Gorongosa National Park has been part of a restoration agreement signed in 2008 between the Carr Foundation and the Mozambican government. Among the many species reintroduced, notable predators include leopards (*Panthera pardus*), spotted hyenas (*Crocuta crocuta*), African wild dogs (also known as painted wolves, *Lycaon pictus*), and golden jackals (*Canis aureus*). These carnivores were firstly introduced in 2018, 2022, 2018, and 2022, respectively. Currently, seven leopards, 19 hyenas, seven golden jackals, and more than 200 wild dogs are present in the park (data from Gorongosa annual reports). Wild dogs were first introduced in 2018 and then again in 2019 from South Africa. The Park started with the introduction of these carnivores after the period of this study. According to the data from camera traps and direct observation, some hyenas have already started repopulating the park.

By 2016, the carnivore team, under the Restoration Project recovered the lion population from below 20 to about 104 (Bouley et al. 2018) and more than 200 in 2025. Before the Civil War, lions and wild dogs were the main ecological controllers of the herbivores (Bouley et al. 2021).

### Data collection

#### Bone surveys

Bone collections were carried out from 2016 to 2023 using transects, plots and opportunistic methods. Nineteen transects (six in annually and 13 in non-annually flooding landscapes) were carried out in 2017 and 2019. The transects consisted of lines of 5 km in length and 50 to 100 m in width. Ninety-six (96; 32 in annually and 64 in non-annually flooding landscapes) plots were designed and surveyed in 2021 and 2023 to better compare the data with other sources of data such as camera traps and biennial aerial surveys. The opportunistic method consists of surveys by researchers conducting different projects and opportunistically recorded carcasses around places of their data collection. For each specimen, species, sex, age, weathering stage, GPS points, minimum number of individuals (MNI), and minimum number of elements (MNE) were recorded.

### Spatial data extraction and integration

Spatial data for roads, rivers, lakes, and pans were obtained from Gorongosa scientific reports and other publications (Stalmans et al. 2019; Stalmans and Beilfuss 2008). To accurately measure distances and extract environmental data for our analysis, we utilised spatial data from landscape type, roads, rivers, pans, and Lake Urema in GNP. We calculated the minimum distances from each carcass to the nearest road, pan, river, Lake Urema boundary. Relevant shapefiles were imported and transformed using the *sf* package (Pebesma and Bivand 2023), ensuring that all coordinate reference systems matched (CRS = 4127). This approach allowed us to quantify environmental variables critical for understanding the factors influencing primate and non-primate carcass distribution in GNP.

### Data analyses

Using a binary response variable (primate vs. non-primate taxa), we applied Generalized Linear Models (GLMs) to identify key predictors. The best-fit model was selected based on the lowest Akaike Information Criterion (AIC) and VIF (Variance Inflation Factor) using the *car* package (Fox and Weisberg 2019), for removing multicollinear variables. Additionally, we used Fisher’s Exact Tests to investigate whether the bone abundance of primates significantly differs from that of other mammal taxa across annually and non-annually flooding landscapes in GNP. This integrated approach highlights the crucial role of environmental variables in shaping both primate carcass distribution and bone abundance patterns.

All statistical analyses were conducted in R v. 4.4.1 (R Core Team 2024), and RStudio v. 2024.12.0+467, 64 for MacOS (Posit Team 2024) with α set at 0.05. The original code can be found in the repository available in the link: https://github.com/jacintomathe/Mathe_paper_four.git. The spatial data analyses and visualisation were conducted using Quantum Geographical Information System (v. 3.32.2; (Moyroud and Portet 2018) and v. 4.4.1 (R Core Team 2024).

## Results

**Fig. 3:**
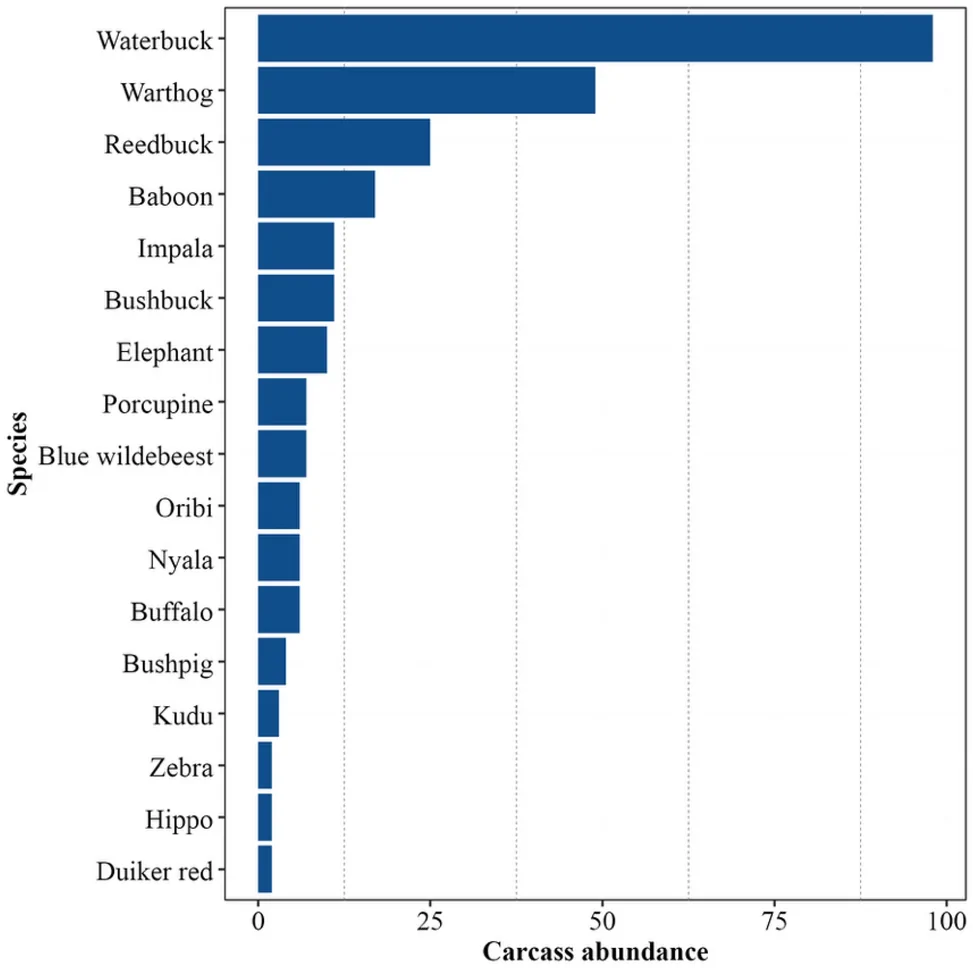
Abundance of mammalian carcasses recorded across GNP

For the analyses, we used 266 specimens representing 18 different mammal species: 17 carcasses of chacma baboons and 249 for the other mammalian taxa documented over a seven-year period in GNP (2016-2023). The complete list of analysed mammalian species and their relative abundance across landscapes of GNP is provided in (Fig. 3).

**Fig. 4:**
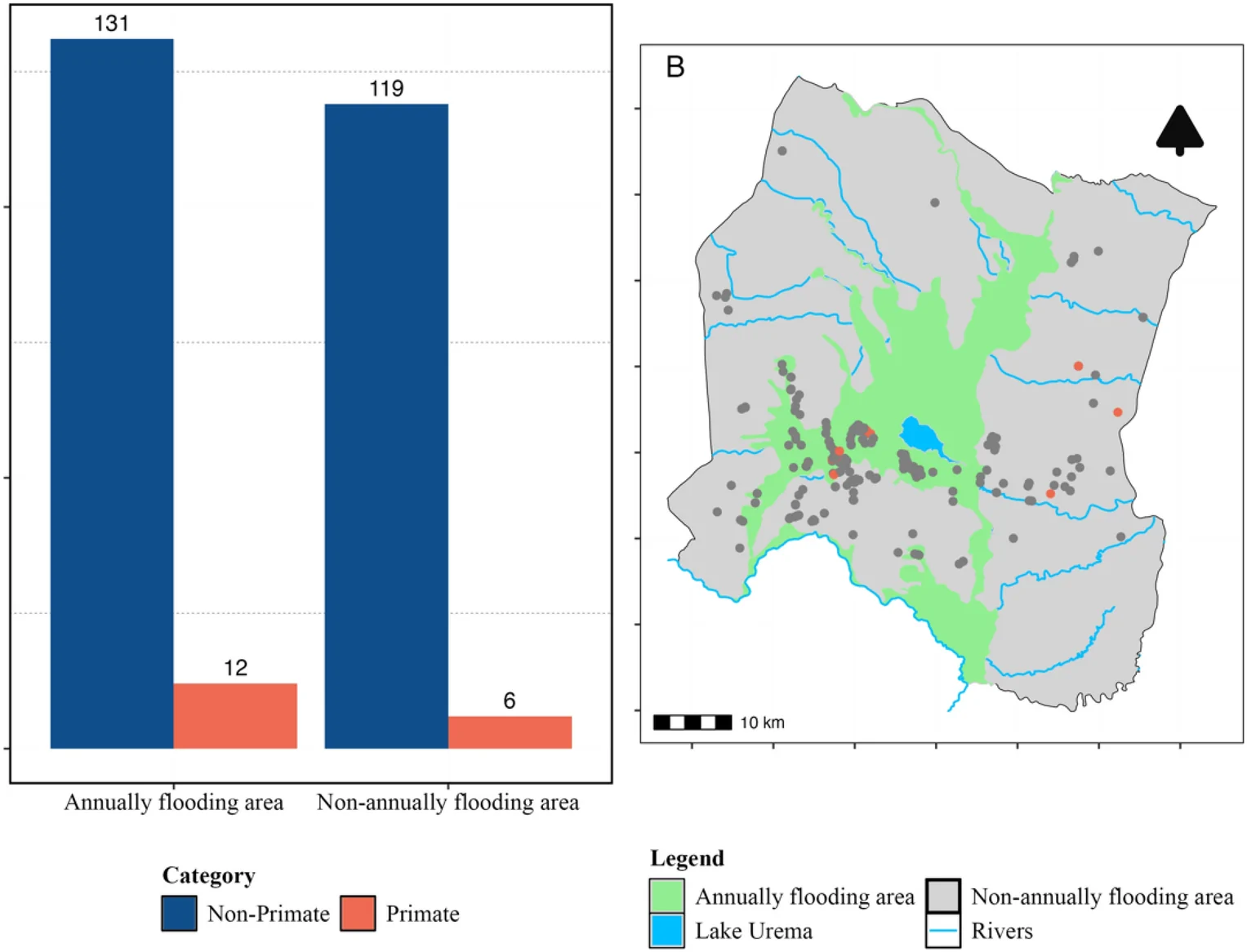
Number of carcasses in annually flooding and non-annually flooding areas (**A**). Map of the distribution of primate and non-primate carcasses in the landscapes of Gorongosa National Park (**B**)

The proportion of primates vs non-primates, displayed in Fig. 4, highlights that there are more non-primates than primates recorded overall (Fig. 4A) in bone surveys in the ecosystems of GNP (Fig. 4B). Primate carcasses in the annually flooding areas are twice as abundant as in the areas that rarely flood (Fig. 4A, B).

### Ecological influence on the likelihood of recording primate carcass

In the Generalized Linear Models (GLMs), although we found that proximity to rivers was not significant (*p* = 0.072 see Table 1), there was a general trend such that close to the rivers there is a slightly higher number of primate bones than further away. Distance to roads and estimated proximity to Lake Urema and pans did not significantly impact primate bone occurrence (*p* > 0.05 see Table 1). Similarly, flooding does not affect primate bone occurrence as bone numbers deposited in flooding landscapes are not significantly different from non-flooding areas (*p* > 0.05). The results further revealed that despite not being significant, proximity to rivers positively affect primate bone occurrence (estimate = 0.12, *p* = 0.072). However, in annually flooded areas, the opposite pattern prevails, proximity to rivers shows negative impact on primate bone occurrence (estimate = 0.135, *p* < 0.05 in Table 1).

### Bone abundance in annually and non-annually flooding landscapes in GNP

**Fig. 5:**
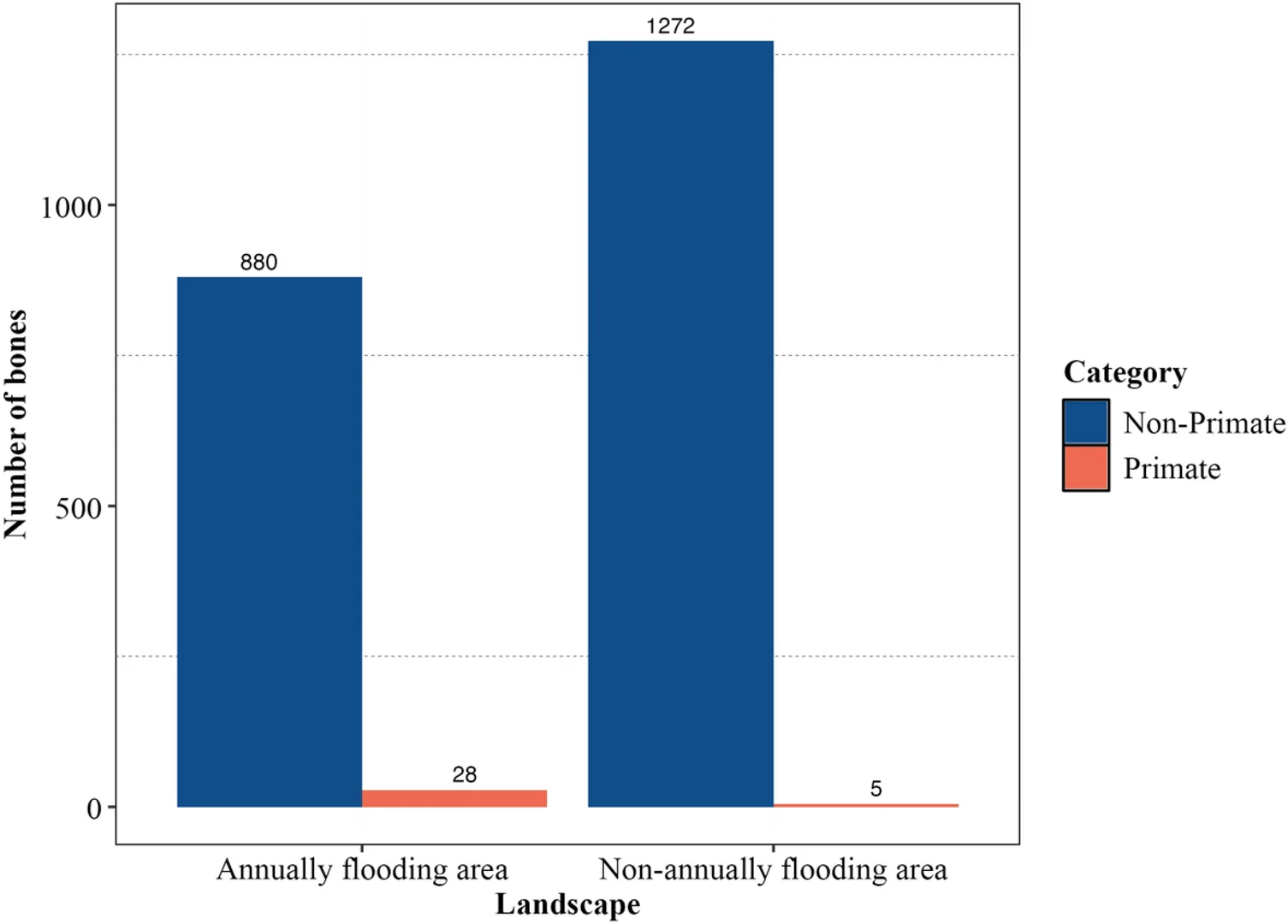
Number of bones of primate and non-primates across the annually and non-annually flooding landscapes in GNP

The results of Fisher’s Exact Test indicate that landscape type (annually flooded vs. non-annually flooded) significantly influences bone deposition for both primates and non-primates (p < 0.05). Annually flooding landscapes harbour slightly higher proportion of primate bones and lower proportion of non-primate bones. In contrast, non-annually flooding landscapes harbour a greater proportion of bones of non-primates than of primates (Fig. 5).

## Discussion

The high representation of larger herbivore carcasses such as waterbuck and warthog in the osteological surveys of GNP, as shown in Fig. 3, may indicate high mortality rates in open landscapes or differential carcass preservation due to body size and predator preferences. The lower representation of primates could be attributed to the resilience of primate species during periods of food scarcity in the long dry seasons. Primates often have access to alternative food sources (Conklin-Brittain et al. 1998; Wrangham et al. 1998) such as fruits and plant bulbs from succulent species, making them less susceptible to starvation, and thus primate death rates may be lower, resulting in fewer bone remains. In contrast, herbivores, which rely heavily on grass and shrubs, face fewer alternatives when these food sources dry out, leading to higher mortality rates. Other factors that may affect primate carcass abundance include ecological influences, which are explored and discussed in further detail below. In addition, the life history of primates may play a significant role. Baboons can live up to 45 years in captivity and 30 years in the wild (Rhine et al. 2000), while vervet monkeys have a maximum lifespan of around 30 years (Kingdon and Hoffmann 2013). In the absence or scarcity of their primary predators, baboons and vervets likely have reduced mortality rates. Currently, in GNP, there is a repopulation of leopards due to reintroductions and new births from reintroduced individuals. Despite this repopulation, the leopard population remains low. Consequently, vervet monkeys and baboons are less abundant in bone assemblages compared to species of similar body mass, such as bushbuck, impala, and oribi, whose remains are almost comparable to their living populations. This discrepancy can be explained by two factors: predation and lifespan. These herbivore species live two to three times shorter lives than vervet monkeys and baboons, making their remains more prevalent in bone collections. Additionally, the primary predators of impala, oribi, and bushbuck are in higher abundance, with an estimated 200 lions in the park according to the latest carnivore team count and aerial survey (Bouley et al. 2018; Stalmans and Peel 2022).

Furthermore, the birth rates and lower death rates of these primates contribute to their population growth and stability in the ecosystem. For instance, vervet monkeys typically have a birth rate of around one offspring every 1–2 years (gestation length of 163 days) per female and a death rate of about 2% per year, while baboons have a slightly slower reproduction rate, with females giving birth approximately every three years (20–28 months of inter-birth interval) (Hill 2000; Kingdon 2013a) and only 3–4% of the population die per year (Rhine et al. 2000). On the other hand, impala, oribi, and bushbuck have potentially rapid population growth indicated by shorter gestation periods (less than eight months) and inter-birth intervals below 18 months (Kingdon 2013b) and higher death rate of at least 20% and shorter longevity, 12, 14, and 18 years for bushbuck, oribi, and warthog, respectively (Kingdon 2015). This implies that these herbivores will contribute greater numbers of carcasses to the ecosystem.

The Generalized Linear Models (GLMs) for predicting the likelihood of finding primates over other taxa based on the ecological variables revealed that water bodies, particularly rivers in non-annually flooding areas, have a significant influence. However, other factors do not have a significant influence in this analysis. Our analysis indicates that in contrast to other taxa, primate remains are preferentially accumulated closer to water bodies. Water bodies may be avoided by primates as a strategic adaptation to minimise predation risk. Rivers, pans and lakes are known to be ambush sites for predators such as lions and wild dogs, especially during the dry season when these water sources become critical for survival (Hutson 2016). Furthermore, primates are less water-dependent than other species, particularly grazing species such as oribi, waterbuck, and impala, which likely contributes to the lower abundance of primate carcasses near water bodies—areas known to be high-risk for predation (Hill 2000; Kingdon 2013a, b), they cross rivers, especially when water levels are lower during the dry season (Lewis-Bevan et al. 2025). During the river crossing, they are more likely to be caught by crocodiles, which camouflage themselves under mud and plants (unpublished camera trap videos) (Carter et al. 2020). These observations of individuals crossing rivers, which contribute to the higher risk to be caught by predators such as crocodiles underscores the importance of seasonal mobility and expanded home ranges in shaping mortality risk. This pattern—where floodplain river crossings increase vulnerability to predation—offers a valuable framework for interpreting the relationship between seasonality, ecology, and the occurrence of hominin remains in the fossil records, particularly regarding the extent of carnivore damage and predation signatures observed in the palaeoanthropological record of eastern and southern Africa.

**Fig. 6:**
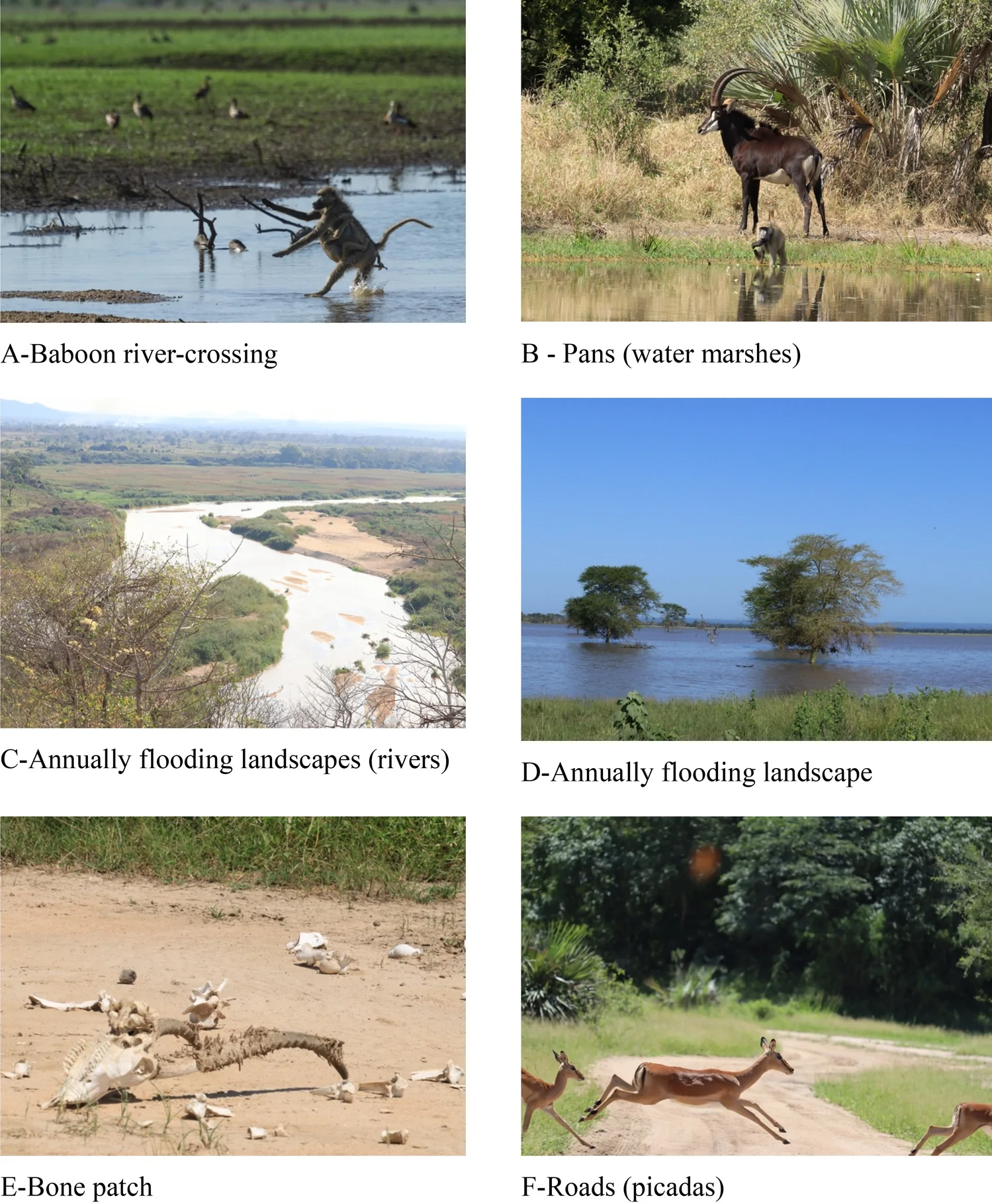
A female baboon ventrally carrying a juvenile jump to cross the shallow Mussicadzi River, Photo: Lee Bennett, 2018 (**A**). A pan during the rainy season with a baboon and a sable antelope (**B**). Pùngué river during the transition from dry to rainy season (**C**). Floodplain during the peak of rainy season (**D**). Bone patch on sodic soil during dry season (**E**) and roads (picada 3) at the end of rainy season (**F**)

A higher abundance of primate carcasses near rivers than in areas further away is supported by the higher number of carcasses found in flooding regions of GNP, which is twice as many as those recorded in areas that do not experience annual flooding. These observations may be linked to the behaviour of primates during the change of the season, particularly from dry to rainy seasons. Primates living in the floodplain tend to have larger home ranges compared to those in woodland areas (Hammond et al. 2022; Lewis-Bevan et al. 2025). Expanding their home range requires considerable effort, as flooding forces them to cross rivers such as the Mussicadzi, Sungue, and Urema in search of safer areas. Previous studies have shown that during the rainy season, baboons in floodplains move towards the southern part of the park, which is elevated and drier (Hammond et al. 2022). During this movement, a significant number of individuals, particularly the infected and weaker ones, may perish (Fig. 6).

Baboons in the park face a range of natural threats, with leopards being their primary predators. However, the low abundance of leopards in the area suggests that other factors may contribute more significantly to mortality. Notably, crocodiles, which have been observed to move up to 300 m from the water (field observation from camera traps videos), may play a key role in the distribution of primate carcasses. This suggests that although some baboons may die from injuries or infections away from water sources and subsequently carried to rivers or Lake Urema by crocodiles. The observed accumulation of primate carcasses near water could also be influenced by a variety of other factors. Changes in baboon home ranges during the rainy season could lead to higher mortality in flooding areas, where conditions are harsher and movement more difficult (Beardmore‐Herd et al. 2025).

The findings of this research also provide a foundation to test whether ecological influences vary by species group in the fossil record. For example, some fossil sites, such as the Mursi Formation in the lower Omo Valley of Ethiopia, contain few primate fossils. This scarcity may not only reflect the absence of leopards that preferentially targeted primates but could also result from taphonomic factors—such as bone decomposition and preservation conditions—that were unfavourable for primate remains. Observations from Gorongosa indicate that baboons and vervet monkeys carry deceased group members to locations that have not yet been identified (Video available here), these observations suggests that similar behaviours in the Mursi palaeoenvironment may have influenced bone distribution. If primates at Mursi dragged carcasses into habitats with higher soil pH or more acidic conditions (Behrensmeyer 1978), this could have accelerated bone decay and reduced the likelihood of fossil preservation. However, bone preservation may vary under different conditions, with higher-density contexts generally associated with a greater likelihood of preservation (Lam and Pearson 2005).

Future research could focus on tracking fresh carcasses within the floodplain, using GPS devices attached to them and increasing the number of collared baboons in the floodplain. This approach would provide insights into baboon movement patterns across different seasons and allow for a more detailed understanding of how environmental factors influence mortality and carcass movement. Seasonal bone surveys in the same baboon home range could complement this data, shedding light on seasonal carcass accumulation over time. Moreover, long-term monitoring of the two baboon troops studied previously would help assess population turnover and identify the primary causes of mortality under varying environmental conditions. This research could provide valuable information for conservation efforts, particularly in understanding how different species interact with their environment and the factors influencing their survival.

The lack of a significant role for the distance to roads in impacting primate carcass occurrence contradicts the initial predictions and suggests that these factors are less critical in shaping primate bone distribution in GNP. The minimal impact of these variables highlights the unique role that water bodies play in the spatial patterns of primate carcasses. This primate bone scarcity may also be related to the fact that leopards are in low abundance in GNP having been detected only once via camera traps. In other African ecosystems, leopards (*Panthera pardus*) are well-documented as primary predators of baboons, highlighting a predatory relationship that can significantly influence primate populations. For example, in Moremi Game Reserve, Botswana, leopards frequently prey on chacma baboons (*Papio ursinus*), utilising ambush tactics that exploit the dense vegetation and open water sources where baboons gather, making them accessible despite their social vigilance (Busse 1980). Similarly, studies in the Ngorongoro Conservation Area, Tanzania, show that leopards opportunistically target baboons, particularly during periods of food scarcity, when they become a key dietary component (Bertram 1982).

In Phinda Private Game Reserve, South Africa, leopards are the main natural predators of baboons, with observed hunting strategies adapted to the savanna-woodland mosaic that characterises this region (Balme et al. 2007). Here, leopards rely on their stealth and climbing ability to capture baboons near their roosting sites. Additionally, Mahale Mountains National Park, Tanzania, provides another example of leopards preying on olive baboons (*Papio anubis*), often targeted when moving on the ground or away from their sleeping trees (Nishida et al. 1979).

These examples illustrate that, in areas like Moremi, Ngorongoro, Phinda, and Mahale, leopards occupy a crucial ecological niche as primary predators of baboons. The abundance of leopards in these areas correlates with notable impacts on baboon populations and carcass availability, particularly in habitats where baboons constitute the primary prey of leopards. In contrast, GNP lacks a sufficient predator population to influence primate carcass abundance, highlighting the distinct ecological dynamics in GNP relative to other African ecosystems.

Primates often live in social groups that provide several benefits, including enhanced vigilance and collective defence mechanisms. These social structures likely contribute to the lower predation rates for primates enhancing lower bones recorded in the landscape. However, other causes of death, such as infanticide and secondary infection of injured individuals, may maintain population stability (Kingdon and Hoffmann 2013).

This analysis underscores the importance of specific environmental features, particularly water bodies, in understanding primate and non-primate carcass occurrences in GNP. These findings provide valuable insights for conservation and management strategies, emphasizing the need to consider species-specific behaviours and ecological needs. By focusing on the unique role of water bodies and the adaptive strategies of primates, conservation efforts can be better tailored to protect these species and maintain the ecological balance in GNP.

Fisher’s Exact Test indicates significant differences among landscapes of GNP concerning the bone abundance of primates with non-flooding landscapes showing slightly higher occurrence of non-primate and lower primate bones. Conversely, annually flooding landscapes harbour a higher density of primate bones and a lower density of non-primate bones. This could be explained by the methodological approaches employed, as the osteological surveys are affected by visibility, accessibility by road and sampling effort. The flooding landscapes (Rift Valley Riverine & Floodplain) are more open and provide higher visibility for osteological surveys (Stalmans & Beilfuss 2008), and higher numbers of primate carcasses were found in these landscapes. Flooding landscapes are also more accessible by road (Stalmans & Beilfuss 2008), which facilitates osteological surveys. However, the sampling effort was higher in non-annually flooding landscapes (13 transects and 64 plots) than in annually flooding landscapes (6 transects and 32 plots).

In general, the low abundance of primates in the Gorongosa bone assemblages, despite their high abundance in aerial surveys, suggests that their scarcity or absence in the fossil record does not necessarily indicate a true absence or low population in past landscapes that may have been geologically and ecologically similar to Gorongosa National Park (Bobe 2011; Bobe and Reynolds 2022). Instead, it may reflect that primates were differently affected by taphonomic processes and ecological and demographic factors that influenced the preservation of other taxa. This underscores the importance of targeted sampling strategies to optimise the discovery of primate fossils.

From these insights, one could further this research by increasing the sampling effort targeting the analysed features (lakes, rivers, pans, and roads) and applying the modern technology to track carcasses in both type of landscapes to further understand other variables that may influence bone deposition in GNP. For instance, baboons have been reported to carry their infants after death, but there is not report of accumulating them in specific locations, but it could happen that the places they are left were not targeted in this study and or are not accessible, but with the application of for example GPS tracking devise, one would understand where they are mostly and perhaps be found, which would contribute to some adjustment to the methodology applied in this study. Apart from increasing the sampling effort, one could also consider increasing the sample size and analysing the data using the same approaches to better understand the fossilization process under different terrain conditions and the ecology of GNP.

## Conclusion

The bone assemblage in Gorongosa National Park reveals a disparity: the number of baboons in the assemblage does not reflect their abundance in the living community. Our results show that primate carcasses are more frequently found near rivers, while non-primate carcasses tend to accumulate further away from these water bodies. Furthermore, our findings indicate that the distribution of primate bones differs significantly from that of other mammals, with the higher number of primate bones observed in the annually flooding landscapes than in non-flooding landscapes. These findings challenge assumptions about the scarcity of primate fossils at some palaeontological sites like the Mursi Formation. Rather than signifying a low abundance of primates in these ancient ecosystems, such patterns may instead reflect ecological factors—particularly the role of water sources—influencing fossil preservation and accumulation. Further studies should also focus on automated detection methods (e.g., camera traps and GPS tracker devices) to examine crocodile and other predator interactions with primate carcasses, as well as primate involvement in carcass distribution and bone deposition. Additionally, further studies with increased sample size would shed light on the variables that contribute to primate carcass absence or rarity in some ecosystems where living populations are known to exist. These research outcomes have implications for our understanding of primate bone accumulations in the African fossil record.

## Supplementary Information

Below is the link to the electronic supplementary material.

Supplementary file1 (DOCX 17 KB)
