# Supplementary material for: Primate carcass ecology in the Urema Rift, Mozambique
Source: Primates. 2026 Jul 20;67(5):579–92. doi: 10.1007/s10329-026-01275-y (PMC13541799; doi:10.1007/s10329-026-01275-y)
Supplement: Supplementary file 1 [file 10329_2026_1275_MOESM1_ESM.docx]

# Supplementary materials

Table 1. GLM outputs of the Influence of ecological variables on the likelihood of recording primate carcasses. In blue are the significant GLM outputs.

| term | estimate | std.error | statistic | p.value |
| --- | --- | --- | --- | --- |
| (Intercept) | 0.03 | 1.283 | -2.72 | 0.006 |
| landscapeNon-annually flooding area | 1.073 | 1.474 | 0.048 | 0.962 |
| mDist_to_UREMA | 2.627 | 0.96 | 1.006 | 0.314 |
| mDist_to_road | 0.863 | 1.706 | -0.087 | 0.931 |
| mDist_to_gnp_pans | 1.654 | 0.703 | 0.716 | 0.474 |
| mDist_to_river | 0.135 | 1.115 | -1.798 | 0.072 |
| landscapeNon-annually flooding area:mDist_to_UREMA | 0.158 | 1.208 | -1.529 | 0.126 |
| landscapeNon-annually flooding area:mDist_to_road | 1.032 | 1.766 | 0.018 | 0.986 |
| landscapeNon-annually flooding area:mDist_to_gnp_pans | 1.688 | 0.972 | 0.539 | 0.59 |
| landscapeNon-annually flooding area:mDist_to_river | 13.246 | 1.256 | 2.057 | 0.04 |
